# Applied machine learning and artificial intelligence in rheumatology

**DOI:** 10.1093/rap/rkaa005

**Published:** 2020-02-19

**Authors:** Maria Hügle, Patrick Omoumi, Jacob M van Laar, Joschka Boedecker, Thomas Hügle

**Affiliations:** r1 Department of Computer Science, University of Freiburg, Freiburg, Germany; r2 Department of Diagnostic and Interventional Radiology, Lausanne University Hospital, and University of Lausanne, Lausanne, Switzerland; r3 Department of Rheumatology, University Hospital Utrecht, Utrecht, The Netherlands; r4 Department of Rheumatology, Lausanne University Hospital, and University of Lausanne, Lausanne, Switzerland

**Keywords:** machine learning, neural networks, deep learning, rheumatology, artificial intelligence

## Abstract

Machine learning as a field of artificial intelligence is increasingly applied in medicine to assist patients and physicians. Growing datasets provide a sound basis with which to apply machine learning methods that learn from previous experiences. This review explains the basics of machine learning and its subfields of supervised learning, unsupervised learning, reinforcement learning and deep learning. We provide an overview of current machine learning applications in rheumatology, mainly supervised learning methods for e-diagnosis, disease detection and medical image analysis. In the future, machine learning will be likely to assist rheumatologists in predicting the course of the disease and identifying important disease factors. Even more interestingly, machine learning will probably be able to make treatment propositions and estimate their expected benefit (e.g. by reinforcement learning). Thus, in future, shared decision-making will not only include the patient’s opinion and the rheumatologist’s empirical and evidence-based experience, but it will also be influenced by machine-learned evidence.

**Key messages**

- Deep learning can assist rheumatologists by disease classification and prediction of individual disease activity.
- Artificial intelligence has the potential to empower autonomy of patients, e.g. by providing individual treatment propositions.
- Automated image recognition and natural language processing will be likely to pioneer implementation of artificial intelligence in rheumatology.

## Introduction

Most rheumatic diseases have chronic, fluctuating courses that involve complex underlying pathophysiology, which complicates their therapy. Despite the advent of targeted biological and synthetic treatments, sustained remission of rheumatoid arthritis (RA) is achieved in only a minority of patients. For many other rheumatic diseases, such as osteoarthritis (OA), lupus or Sjögren's syndrome (SS), controlled clinical trials for new therapies have been broadly disappointing owing to different disease phenotypes. Perhaps the major current unmet clinical need for RA patients, but also for those with other rheumatic diseases, including OA, is a personalized treatment approach. Given the data-intensive studies necessary to find the best treatment strategies for individual patients, artificial intelligence (AI) can play an important role in the development of personalized medicine. In particular, machine learning (ML), a subfield of AI, can foster personalized treatment by providing computers with the ability to learn from experience without rules explicitly specified by humans. The potential for ML in medicine is vast and, compared with conventional statistics, ML offers a plethora of new possibilities.

Although there is a significant overlap in methods between statistics and ML, the application goals [1] and scalability of solutions are generally different. Conventional statistics has a strong focus on accurate summaries of data samples, understanding statistical relationships between variables and correctly estimating population parameters [2]; the main goal of most ML methods, in contrast, is predictive performance on unseen data. Additionally, ML algorithms can automatically learn useful data representations and deal with different sorts of input data, e.g. patient cohorts, medical images and genetic information. Thus, ML fills a significant gap in learning from clinical experience. Ideally, it translates the knowledge gained into clinical evidence, with computers being capable of predicting clinical outcomes, recognizing disease patterns, detecting disease features and optimizing treatment strategies.

Deep learning [3], a specialized subfield within ML that relies on large neural networks, has shown dramatic successes over the past 10 years, driven by increased computational power and massive datasets. Deep learning has shown striking advances in processing and understanding data, including text [4], speech [5] and images [6]. Machine and deep learning are increasingly applied in medicine (e.g. for assistance in medical imaging [7], brain stimulation devices [8] or cancer prognosis [9]). In its first prospective clinical trials, decision-making based on ML was superior to decision-making by physicians alone in the treatment of intensive care patients [10]. Besides its potential to increase the quality of treatment, medical ML also has the potential to increase the quality of care and reduce costs. The aim of this article is to explain the basics of ML and its present and future applications in the field of rheumatology.

## Artificial intelligence and machine learning

**Fig. 1:**
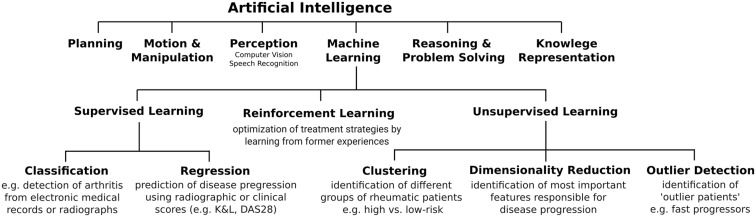
Cognitive capabilities of artificial intelligence and types of machine learning

Artificial intelligence is a subfield of computer science devoted to providing computers with capabilities for intelligent problem solving (i.e. to solve complex problems in a way that we would consider as smart). These capabilities include planning, reasoning, perception or learning (Fig. 1). Machine learning, a subfield of AI, provides algorithms (sequences of well-defined computer instructions that solve a specific problem) that build mathematical models based on sampled data. These mathematical models (called functions) map input data to desired outputs. Inputs can be images and an arbitrary sequence of numerical or categorical data. The selected inputs are later referred to as input features.

**Fig. 2:**
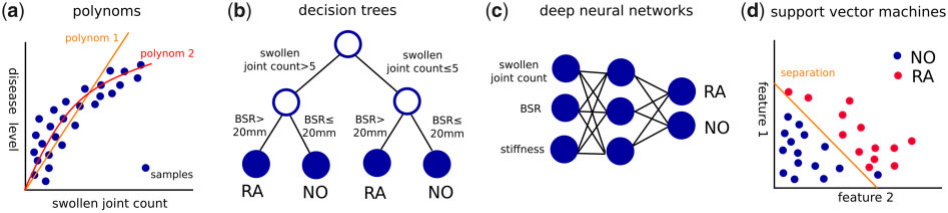
Machine learning models use different function representations to map input features to certain outputs

To represent the mapping, different functional representations can be used (called models), such as polynomial functions, decision trees [11], support vector machines (SVMs) [12] and artificial deep neural networks (Fig. 2). Linear functions cover linear relationships between input features and a continuous output variable. Decision trees are tree-like models of decisions and their possible consequences. An improvement of decision trees is random forests [13], which are ensembles of decision trees that classify samples by a majority vote of all trees. The use of ensembles leads to a lower variance and lower bias. SVMs are trained to find the best possible separation of different categories by adapting weights of polynomial functions. Another method, called the *k*-nearest neighbour approach, classifies samples by a majority vote, assigning the class most common among the *k* samples with the most similar features.

The above-mentioned models are built or trained in different ways. During training, most of the ML models are adapted by calculating the error of the model outputs with respect to the desired targets and adapting the model parameters so that the error is minimized. It is important not only to learn the samples by heart but also to be able to detect hidden patterns and rules in order to generalize to new, unseen data. In order to evaluate the quality of the models, datasets are split into different parts, where the largest part of the data is used for training (training set). The rest of the data are used to evaluate the performance (validation and test sets). In cross-validation methods, *k* different parts of the data are held out during training and are only used afterwards to assess generalization of the trained model to data not seen during training. All validation results are combined for a more robust assessment of the model performance to avoid selection bias and overfitting. The test set is used only once, for a final, unbiased performance evaluation. The performance on a test set can be evaluated using different metrics, such as accuracy, sensitivity, specificity and area under the curve (AUC) for classification tasks and the mean squared error for regression tasks.

The data used to train ML systems are usually real data. ML systems can also be trained with artificial data collected from simulators, where actions can be taken by trial and error to learn about different outcomes. In the fields of robotics, games or autonomous driving, simulators can be used to expose ML methods to a large number of new situations [14, 15] during training. In medicine, ML systems are mainly trained and evaluated on historical datasets. In cases showing convincing performance, they can then be evaluated on real control groups using rigorous safety precautions and ideally as a controlled clinical trial.

Machine learning consists of the subfields supervised learning, unsupervised learning and reinforcement learning (Fig. 1). Deep learning, another subfield of ML, expands the capacity of all these areas by using artificial deep neural networks to map input data to desired outputs.

### Deep learning

**Fig. 3:**
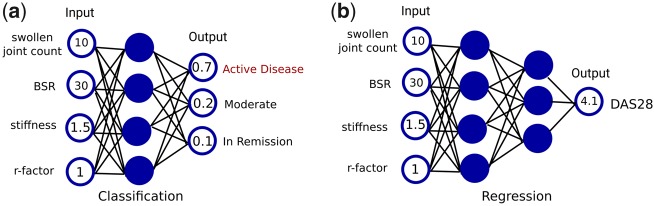
Difference of classification and regression models for disease prediction in RA

In deep learning, data representations are learned automatically by deep neural networks. Deep neural networks can learn highly complex, non-linear mathematical functions. They consist of several sequential layers composed of many simple non-linear operations [3], so-called neurons. Historically, these operations were loosely inspired by (simplified) information processing principles in biological neurons: input signals coming from dendrites are integrated in the cell body, and once the membrane potential of the cell exceeds a certain threshold, it generates an action potential that is relayed to other connected neurons via its axon. In an artificial neural network, input features are fed into the first layer of neurons and are then propagated through the network to the output layer. Deep neural network architectures can deal with different types of input data (e.g. medical images, text or any other type of patient data). There exist different architectures for different types of input data, {e.g. fully connected neural networks (shown in Fig. 3), convolutional neural networks [3] and recurrent neural networks [16]}.

Convolutional neural networks are typically used for images and other data with a grid-like structure. They perform a mathematical convolution operation on every part of their inputs to learn increasingly abstract features in multiple layers. A typical example can be seen when convolutional neural networks are trained on natural images. The features that arise through the training process in the first layers usually specialize in detecting contours and edges, and features in later layers are able to combine these earlier features to detect more complex objects, such as joints or hands. To increase interpretability, convolutional neural networks can be used to generate heatmaps, highlighting areas by colour that contribute most to the decision of the network. Thus, abnormal areas, such as structural damage, can be visualized for diagnostic use.

Recurrent neural networks, such as long short-term memories (LSTMs) [17], are a class of artificial neural networks with an internal memory to process sequences of data, such as handwriting, speech or numerical time series. In the field of medicine, LSTMs are used e.g. for outcome prediction in intensive care units [18], heart failure prediction [19] or prediction of health-care trajectories from medical records [20].

### Supervised learning

**Fig. 4:**
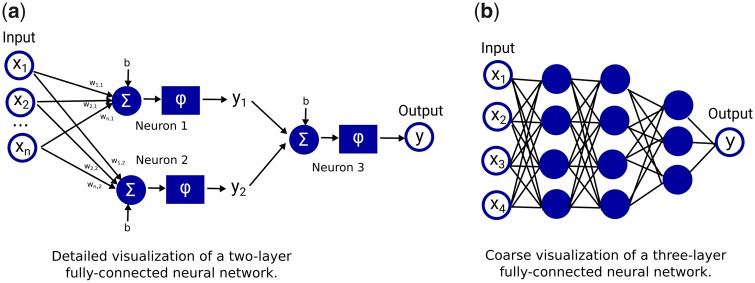
Visualizations of fully connected neural networks

In supervised learning, machine learning models are trained based on given examples, consisting of inputs and desired outputs provided by an expert (e.g. the rheumatologist). Outputs can either be a set of categories (e.g. active disease, moderate or in remission) or they can be numbers (e.g. the absolute DAS28 score). Models trained to output a choice of categories (e.g. low, moderate or active disease level) are called classification models. Models that are trained to output real numbers are called regression models. The difference between classification and regression is shown in Fig. 4 for the example of prediction of disease levels.

**Fig. 5:**
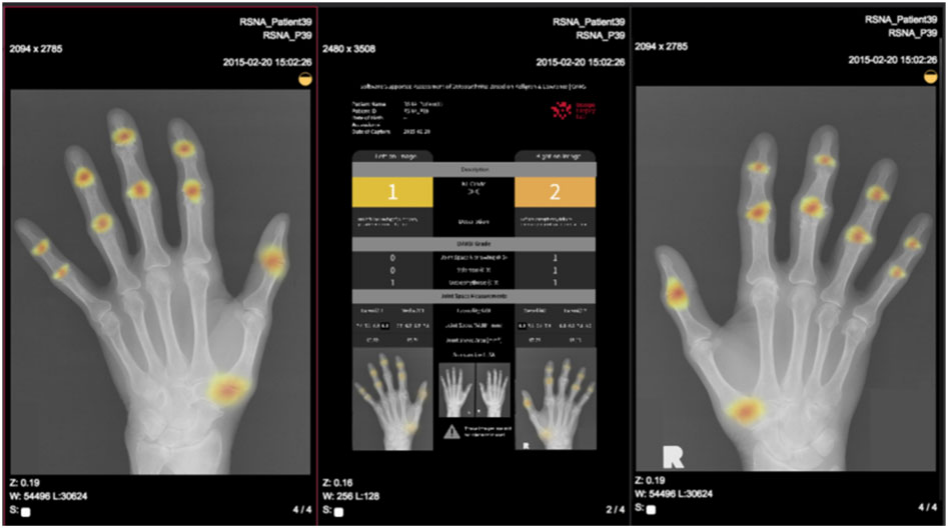
Heatmap of a hand radiograph indicating regions of high attention for OA Courtesy of ImageBiopsy.

### Unsupervised learning

Unsupervised learning aims to find as-yet unknown underlying patterns or structures in unlabelled data. Unsupervised learning methods are often used to process large databases, such as electronic medical records (EMRs) or large patient cohorts. They can also cluster patients (subdividing them into groups) and characterize outliers or other important features. Popular models include hierarchical clustering [21] and *k*-means [22]. Furthermore, they can be used to reduce the dimensionality of the given data (e.g. with principle components analysis [23] or with unsupervised deep learning models, such as Autoencoder networks [24]). This can help clinicians to gain a better understanding of situations, visualize relationships in the data and focus their practice on a particular disease feature.

### Reinforcement learning

In reinforcement learning (RL) [25], computers learn strategies on how to act optimally in certain situations with respect to a given criterion (e.g. expected improvement of health). An RL algorithm obtains feedback on how well it is performing as measured by this criterion through reward values during training. In the field of rheumatology, RL could be used, for example, to optimize treatment strategies. Based on the observed data, a function can be learned that specifies how valuable a certain treatment is expected to be in the future. This value function can be represented by regression models, such as regression trees or deep neural networks. The strategy can then be extracted from this function by taking the best treatment with the highest expected return.

## Application of machine learning in rheumatology

### Electronic diagnosis

Online electronic diagnosis systems, also called symptom checkers, are increasingly used by the population and, to a lesser extent, by health-care professionals. Most symptom checkers are ruled-based systems, based on simple (non-machine-learned) decision trees. Currently, >100 general symptom checkers exist. They are usually based on textbook knowledge, where one or multiple symptoms lead to a particular differential diagnosis. A survey showed that in-two thirds of cases, the correct diagnosis was among the top 20 diagnoses given. The correct diagnosis, however, is provided in only around one-third of cases and inflammatory arthritis is correctly diagnosed in <20% [26]. Symptom checkers are now becoming more advanced in how they communicate with patients. Chatbot symptom checkers can process and respond to user input interactively (e.g. in text or audio chats). Applications such as symptomate.com use chatbots for e-diagnosis (https://symptomate.com/chatbot/). Symptom checkers become more powerful as they shift from simple rule-based systems to experience-based, deep learning systems that can exploit collected data (e.g. clinical features, age, imaging). A press release from the Isabel symptom checker and triage tool (https://symptomchecker.isabelhealthcare.com/) proclaimed that its ML used data from >6000 cases. Unfortunately, to the best of our knowledge, no information on the training data and diagnosis validation process has been published for this tool. For supervised diagnosis systems, diagnoses validated by medical doctors are necessary to build up a robust, labelled training set. In cooperation with IBM Watson (https://www.ibm.com/watson), Versus Arthritis has launched a cognitive virtual assistant to provide personalized support for self-management topics, such as medication and exercise (https://www.ibm.com/case-studies/versus-arthritis).

### Disease detection and stratification

Machine learning is increasingly applied to EMRs in various medical fields [27], because they contain large, heterogeneous datasets that can be used to train disease detection or classification approaches using supervised learning methods. To date, many of the disease detection approaches in rheumatology have used SVMs or random forests and are performing classification (which means assigning categories, e.g. RA/non-RA, see Supervised learning section).

Carroll *et al.* [28] used SVMs to detect patients with RA in an EMR by investigating the billing codes and medication exposure. With a very small dataset of ∼100 training samples, they achieved an excellent AUC score of 0.97. Lin *et al.* [29] proposed an automatic approach for detecting RA disease activity from an EMR. Different machine learning models were trained on a training set of >2500 clinical notes and laboratory values and then evaluated on two test sets totalling >2000 notes. The models extracted terms such as synovitis, pain or stiffness as input features by using a text analysis tool on clinical notes, and they also used laboratory values of CRP or ESR. With this clinical and laboratory information, the disease activity of each RA patient was predicted using the following categories: high (DAS28 > 5.1), moderate (DAS28 3.2–5.1), low (DAS28 2.6–3.2) and remission (DAS28 < 2.6). The best SVM achieved an AUC score of 0.831. The authors concluded that automatically discovering RA disease activity from EMR data was, in principle, a learnable task, with results approximating human performance.

Shiezadeh *et al.* [30] performed RA disease detection on a dataset of ∼2500 patients referred to a rheumatology clinic in Iran. They selected 11 features using feature-selection techniques such as the χ^2^ test (significance test). Painful elbow and knee joints, sex, number of affected joints and ESR test results had the most impact on the detection. As an ML method, they used an ensemble of decision trees and compared them with *k*-nearest neighbours and SVM techniques. Their best model yielded 85% accuracy and sensitivity/specificity of 44%/74%.

A study in the UK used random forests to identify RA patients from the clinical codes in an EMR [31]. A random forest identified eight predictors related to the diagnostic codes for RA (i.e. treatment with DMARDs, prednisolone or MTX. With a training set of >15 000 and testing set of >5000 patients from two clinics in Wales, they achieved 92% accuracy, with sensitivity/specificity of 86%/94%.

Chin *et al.* [32] carried out a large-scale, early-disease risk assessment of RA patients using an EMR in Taiwan. The goal of early risk assessment was to discover hidden factors and establish assessment models based on the diagnoses collected before a formal diagnosis. The dataset used 1000 RA and 500 000 non-RA patients, with diagnostic codes as input features and matrix factorization for computing latent risk factors. Using an SVM, they identified early-stage RA with sensitivity and specificity of ∼74 and ∼70%, respectively.

Machine learning was applied to a cohort of 120 SLE patients in order to detect erosive arthritis [33]. Using logistic regression, they achieved an AUC of 0.8. Feature-importance analysis showed a relatively high importance of >40% for anti-CarP antibodies and >30% for anti-CCP antibodies. In a recent study, SLE patients were identified efficiently in EMRs based on natural language processing and logistic regression using ICD-9/10 codes with a specificity of 97% and a positive predictive value of 90% [34].

### Prediction of disease progression

Given that long-term disease development can be influenced by many unforeseen factors, prediction of disease progression is much more sophisticated than disease detection. However, ML can be used to learn disease prediction models, which are of great interest for treatment choice or consultation intervals. In 2018, EMR data were used in The Netherlands to predict flares in RA patients and to steward treatment tapering [35]. A flare was defined as an increase of DAS28 and swollen joint count or an increase in medication from the last visit. Demographic, laboratory and medication data along with clinical data (follow-up time, DAS28 and swollen joint count) were used as input features. In 314 patients, they trained different ML models, i.e. random forests, logistic regression and *k*-nearest neighbour models. The best performance was achieved by a random forest, predicting flares with a mean AUC of 0.8.

A mortality prediction model based on a random survival forest [36] (variant of random forest) was used by Lezcano-Valverde *et al.* [37] to predict the mortality of patients, using a training set of a cohort with >1400 patients from a clinic in San Carlos and a validation set of 280 RA patients from a clinic in Madrid. The survival tree identified five mortality risk groups. For 1 and 7 years of follow-up, a sensitivity of 0.79–0.80 and specificity of 0.43–0.48 were reached.

In a recent paper from 2019, deep learning was applied to forecast RA disease activity in 820 patients using data from a US EMR [38]. For disease prediction, the disease activity [clinical disease activity index (CDAI)] was translated into a binary disease activity state consisting of remission/low (CDAI ≤ 10) or moderate/high (CDAI >10). The features considered were demographics (age, sex and ethnicity), prior CDAI score, ESR and CRP level, DMARDs, oral and injected glucocorticoids and autoantibodies (RF and/or ACPAs). The authors reported an AUC of 0.91 in a test cohort of 114 patients. CDAI was the most important feature for prediction of disease, followed by cortisone injections and CRP. Individual DMARDs were less important for prediction of disease activity, potentially owing to the low number of patients and eight different DMARDs.

In a dataset of 1892 RA patients, Guan *et al.* [39] used a regression model based on Gaussian processes [40] to predict the response to anti-TNF therapy after MTX failure, taking into account demographic and clinical data in addition to genetic data (single-nucleotide polymorphisms). Here, the model classified the response to anti-TNF treatment with 78% accuracy. In a Swedish RA registry with 300 patients, three distinct patient subgroups (low, median and high) of persistent pain intensities were identified using unsupervised learning. Additionally, a random forest was used to find predictive parameters among 21 different demographic, patient-rated and objective clinical factors and to patient pain-related subgroups. Using disease severity, swollen joint count and tender joint count acquired as features, an accuracy of 59% was achieved for 3 month predictions.

Hügle *et al.* [41] used a novel dynamic deep neural network, designed for multimodal clinical data, to predict the disease progression to follow-up visits in RA patients. The dynamic deep neural network architecture outperformed random forests and fully connected neural networks, achieving a mean squared error of 0.9, which corresponds to an error of 8% in the range of the target value (change of DAS28-BSR).

### Genetic and transcriptomic biomarkers

In oncology, gene mutations play an increasing role in treatment choices (e.g. in the form of companion drug use). Genetic background is also of growing importance in rheumatology, although clinically gene-expression patterns seem more significant in chronic inflammation (e.g. IFN or other cytokine signatures). Given the technical progress, in the future, genetic and transcriptomic types of data will probably be taken into account for disease classification or treatment choice. Datasets containing DNA or gene expression data could be used to develop new biomarkers, and large, heterogeneous genetic or epigenomic datasets could be used to find new disease patterns and abnormalities.

In 2019, the genetic markers of 7000 psoriasis patients were used as input for ML models to distinguish between patients with PsA and cutaneous-only psoriasis [42]. Using 200 genetic markers, the models identified nine new loci for psoriasis or its subtypes. Prediction of PsA in patients with cutaneous psoriasis was achieved with a remarkable precision of >90% and specificity of 100%.

Microarray-based transcriptome data from synovial tissue were recently used to differentiate between OA and RA [43]. Random forests were used to find the most differential genes by computing the importance of features. With random forests, *k*-nearest neighbours and SVMs, they achieved a very high accuracy of 96%, with a sensitivity of 100% and specificity of 90%.

Machine learning methods were applied to whole-blood transcript data to predict responses to MTX treatment at 6 months after drug initiation in RA patients [44]. ML outperformed statistical models considering clinical covariates only (e.g. baseline disease activity, sex). With regression models such as Ridge regression [45], they achieved an AUC of 0.78 and 0.63 with and without transcript data, respectively.

### Image recognition

Machine learning is increasingly being used for image interpretation in musculoskeletal radiology. Over the last decades, the use of deep learning has increased the performance of image interpretation methods significantly, especially with the development of convolutional neural networks [3]. Automatic image interpretation can serve as a diagnostic aid for physicians in clinical practice e.g. for detection of lesions or regions of high interest by heatmaps (Fig. 5). This is particularly relevant for semi-quantitative or quantitative assessments, which are extremely time consuming (and therefore costly) to perform manually. ML offers the opportunity to provide such quantitative assessment in a more practical, fast and reliable way. In the following paragraphs, examples from the OA literature are given that have been most prolific in terms of the application of AI to rheumatological disorders.

Several methods have been used to detect OA on knee or hip radiographs. Brahim *et al.* [46] used tibial texture analysis to detect OA on 1024 knees in the OA Initiative dataset, achieving 83% accuracy and sensitivity of 87%, with a specificity of 81%. In a study on 420 pelvic radiographs, Xue *et al.* [47] achieved 93% accuracy and a sensitivity of 95% and specificity of 91%, which are results comparable to a senior radiologist.

Although radiography has long been the imaging mode of reference for assessing OA, MRI has now emerged as the mode of reference for assessing all the articular components involved in the development of OA. Liu *et al.* [48] achieved a sensitivity of 81% and specificity of 88% in the automatic detection of femorotibial cartilage lesions using two-dimensional convolutional neural networks. Three-dimensional convolutional neural networks detected menisci and patellofemoral cartilage lesions from MRI datasets [48] with a sensitivity/specificity of 90%/82% and 80%/80%, respectively, comparable to clinical experts [50].

Imaging can also provide a tool to grade the severity of OA (regression, supervised learning). This has typically been done manually by radiologists using semi-quantitative scores, such as the Kellgren–Lawrence (KL) radiographic scoring system or, for MRI, using the Whole Organ Magnetic Resonance Imaging Score (WORMS) or Boston Leeds Osteoarthritis Knee Score (BLOKS). Tiulpin *et al.* [51] used a convolutional neural network to predict five different disease levels (graded according to the KL scale). They achieved 66% accuracy and an AUC of 0.93 (comparable to clinical experts) in predicting the severity of OA among 3000 randomly selected radiographs from the OA Initiative cohort. Furthermore, they used attention maps to highlight the radiological features affecting the convolutional neural network decisions. In a similar study, Norman *et al.* [52] obtained similar results.

Finally, along with morphological datasets, MRI enables the use of compositional techniques, particularly T_2_ mapping, which now enables three-dimensional quantitative assessment of tissue structure [53, 54]. Tools to automate the analysis of compositional imaging techniques and integrate morphological data would be very beneficial to the field of rheumatology. Ashinsky *et al.* [49] showed, with 75% accuracy, that patients progression to symptomatic OA can be detected using T_2_ mapping data.

## Discussion

Machine learning is a young but emerging field in rheumatology [55]. Automated image recognition and scoring lesions on radiographs will probably be some of the first applications assisted by AI to enter routine clinical use. As shown in recent studies, individual disease prediction models will certainly follow. Supervised learning is currently the method most often applied for disease detection and risk assessment in RA. ML models are typically trained using EMR or national cohorts. Here, algorithms can automatically detect and process clinically useful information and can be used for AI-assisted quality control in smaller patient samples (e.g. how many and which patients in your consultations are in remission or not). Although cohorts and local registries offer smaller datasets than EMRs, they are often labelled more specifically and thus enable more sophisticated applications of ML, notably for disease prediction. Using these data optimally, ML applications can help clinicians to gain a better understanding of disease courses and potentially adapt treatments earlier. Current data and our own experience indicate that ML is particularly good at predicting remission, thus helping clinicians to de-escalate or stop treatments and reduce monitoring [35].

**Fig. 6:**
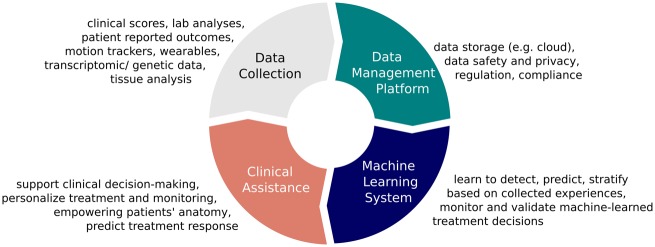
Cycle of artificial intelligence-supported data management and clinical decision-making in rheumatology

In the future, ML will probably also help rheumatologists to find the best patient-specific treatment (e.g. by using reinforcement learning). The more information available, the better ML will perform to support the rheumatologist in disease prediction and treatment choice. Patient-reported outcomes, along with laboratory values, genetic and transcriptomic information (e.g. from synovial biopsies) and radiological data will increase the quality of ML evidence. Once ML learns from its own decisions, we really can speak of AI-supported medicine. To do so, an architecture of data collection, storage, processing, algorithms and, finally, integration in the clinical system and validation of AI support is necessary (Fig. 6). All these elements are important steps to ensure user acceptance, both by physicians and by patients. Although data storage, processing and learning algorithms can be fully automated, data entry, usability and data validation seem to be most crucial.

One of the main challenges for ML applications remains the time for objective clinical assessment (e.g. joint swelling) and data input. In future, whether this is performed purely by the rheumatologist or by specialized nurses and how this process can be automated (e.g. by image recognition) remain to be clarified. Data safety, privacy and compliance are other main challenges. Regulations for ML-assisted applications in the sense of software as a medical device are currently being developed.

We postulate that future shared decision-making processes will be likely to change. Currently, clinical decisions are based on scientific evidence, the rheumatologist’s personal experience and the patient’s preference. Machine-learned evidence could become a cornerstone of shared decision-making in certain situations. We do not think that this new element in clinical decision-making will affect patient–doctor relationships in a negative way. It is more likely, because ML-generated evidence is a neutral tool, that it will empower patients and, to some extent, make them less dependent on the opinion of their doctor.

There are, nevertheless, also technical challenges to overcome before ML can be applied successfully to all medical data; two challenges are the quantity and quality of the data. Deep neural networks, in particular (which are currently the most powerful ML method in many applications), generally require massive training sets. Poor-quality training data (e.g. noisy data, missing values, irregular visits) from EMRs will reduce the overall quality of the model.

Taken together, ML has already shown clinically useful applications in rheumatology. It has the potential to support doctors in clinical and experimental medicine and to foster personalized medicine. For patients, ML offers the possibility of more transparency and autonomy. Integrated databases have the greatest potential to provide sufficient data.

*Funding:* No specific funding was received from any funding bodies in the public, commercial or not-for-profit sectors to carry out the work described in this manuscript.

*Disclosure statement:* The authors have declared no conflicts of interest.
